# Expanding the recognition of monosaccharides and glycans: A comprehensive analytical approach using chemical-nose/tongue technology and a comparison to lectin microarrays

**DOI:** 10.1016/j.bbadva.2024.100129

**Published:** 2024-12-08

**Authors:** Shunsuke Tomita, Chiaki Nagai-Okatani

**Affiliations:** a Health and Medical Research Institute, National Institute of Advanced Industrial Science and Technology (AIST), Tsukuba, Ibaraki 305-8566, Japan; b School of Integrative and Global Majors, University of Tsukuba, Tsukuba, Ibaraki 305-8577, Japan; c Cellular and Molecular Biotechnology Research Institute, National Institute of Advanced Industrial Science and Technology (AIST), Tsukuba, Ibaraki 305-8565, Japan

**Keywords:** Saccharides, Glycans, Optical biosensing, Lectin microarrays, Multivariate analysis, Machine learning

## Abstract

Chemical-nose/tongue technologies are emerging as promising analytical tools for glycan analysis. After briefly introducing the importance of glycans and their analytical methods, including the lectin microarray (LMA) as one of the gold standards, the fundamental principles underlying chemical noses/tongues are explained and various applications for monosaccharides and glycans are introduced. Then, the similarities and differences of these two approaches are discussed. While both technologies aim to comprehensively profile biospecimens based on ‘interaction patterns’ between multiple recognition probes and analytes, each has its own strengths. LMAs excel at specific, targeted analysis based on defined lectin–glycan interactions, whereas chemical nose/tongue offers greater flexibility and expandability in terms of system design, making it well-suited for discovering unknown glycan profiles and detecting broader differences in glycan mixtures. In the future, chemical-nose/tongue technologies may be applied to niche areas in glycan analysis and become powerful tools that complement LMA techniques.

## 1 Introduction

### 1.1 Significance of glycans and glycan analysis in biology and pathology

**Fig. 1:**
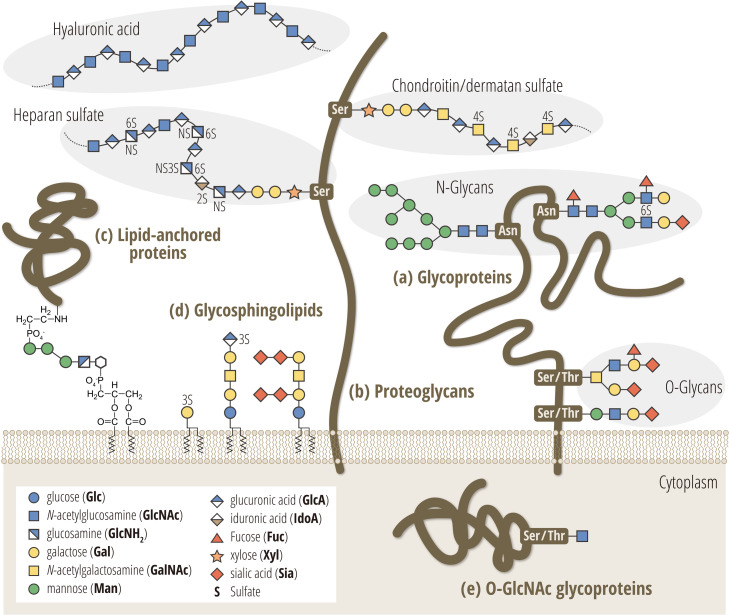
Major classes of eukaryotic glycans. Glycans are generally defined based on the nature of the linkage to aglycones (proteins or lipids). (a) Glycoproteins are complexes in which one or more glycans are covalently attached to a protein, typically via *N*- or *O*-linkages. *N*-Glycans are attached to Asn residues and commonly involve *N*-acetylglucosamine (GlcNAc), while *O*-glycans are linked to Ser or Thr residues mainly via *N*-acetylgalactosamine (GalNAc). (b) Proteoglycans consist of one or more glycosaminoglycan (GAG) chains, such as chondroitin/dermatan sulfate and heparan sulfate, attached to a core protein. Another GAG, hyaluronic acid, typically exists as a free polysaccharide. Some GAGs have sulfated amino or hydroxyl group. (c) Lipid-anchored glycoproteins use a glycan bridge to anchor proteins to the lipid bilayer. (d) Glycosphingolipids (or glycolipids) consist of a glycan attached to the hydroxyl group of a ceramide, typically linked via a glucose (Glc) or galactose (Gal) molecule. (e) Various proteins containing *O*-linked GlcNAc are found in the cytoplasm. Adapted from Fig. 1.6 of *Essentials of Glycobiology, Fourth Edition* (Ref. [2]).

Glycans are a type of carbohydrate chain that, in addition to nucleic acids (genes) and proteins, are known as “the third life chain”. This major biopolymer is expressed in all cells of a wide range of biological species and plays an important role in the regulation of biological phenomena at both the individual and the cellular level [1]. Glycans often exist as glycoconjugates where they are bound to proteins and lipids to form glycoproteins, proteoglycans, glycosylphosphatidylinositol (GPI)-anchored proteins, and glycosphingolipids. In addition, free polysaccharides, such as glycosaminoglycans (GAGs), are also major forms of glycans (Fig. 1) [2]. Glycans play an important role in the functions of the glycoconjugate that they are part of by affecting the physical properties (e.g., stability and charge), interactions with other molecules, and molecular recognition of the glycoconjugate [1]. Many proteins (> 50 %) are glycosylated, and thus protein glycosylation is a major post-translational modification with great significance in protein science. Interestingly, RNA glycosylation has recently been observed at the cell surface [3]. The prevalence of glycans in biological systems underscores the importance of glycan analysis for gaining a better understanding of the molecular mechanisms underlying biological phenomena [1].

Glycan analysis is also important for the application of glycans in medical fields. Given their known involvement in various important biological phenomena, glycans are known to significantly change in structure and quantity depending on the current state of the cell. Accordingly, many diseases such as cancer are accompanied by aberrant glycosylation, which can be used as an indicator of the disease [4]. In the case of glycoproteins, both secreted and membrane-bound proteins are decorated with glycans, and hence can be used for the development of biomarkers and therapeutic targets, respectively [5,6]. In addition, altered glycosylation itself can be a part of the molecular mechanism at the onset and during the progression of diseases [7], and thus the analysis of disease-related glycosylation changes facilitates a better understanding of such pathological phenomena. Furthermore, most biopharmaceuticals are glycoproteins and thus glycan analysis is crucial for quality control and modification of the pharmaceutical functional activities, such as complement-dependent cytotoxicity (CDC) and antibody-dependent cell-mediated cytotoxicity (ADCC) of antibodies [8].

There are many category-specific biosynthetic pathways involving multiple glycosyltransferases, and thus a diverse array of glycans exists [9]. These biosynthetic processes are often complicated because their enzymatic specificities and activities overlap, resulting in multiple enzymes being responsible for a single reaction and a single glycogene being responsible for multiple reactions. In addition, the subcellular localization of these enzymes also affects the structures of the resulting glycans. Hence, it is difficult to infer the structure of a glycan biosynthetic metabolic product only by using gene-expression analysis of the glycosyltransferases. Accordingly, it is indispensable to obtain structural information by analyzing the glycans themselves [10].

### 1.2 General methods for glycan analysis

The structural analysis of glycans is highly challenging compared to that of genes and proteins due to the branched nature of glycan structures and species-specific variations in monosaccharide composition. Accordingly, a variety of analytical methods have been developed to identify and quantify the structure of glycans and their conjugates in biological samples [11]. In general, glycan-analysis methods are specialized according to the type of glycan being examined. For the analysis of glycolipids, liquid chromatography/mass spectrometry (LC/MS) of intact glycoconjugates (without the liberation of the glycan moieties) is commonly used [12]. For the analysis of glycans attached to glycoproteins and proteoglycans, the glycan moieties are typically released from the glycoconjugates and peptide-*N*-glycosidase F (PNGase F) is usually used for the liberation of *N*-glycans. However, there is no established enzyme-based method to comprehensively release glycans from proteins for *O*-glycosylation, and thus chemical methods based on reductive and non-reductive β-elimination are commonly used for this purpose. A common analytical method for the examination of *N*- and *O*-glycans is to label the free reducing ends of the glycans with fluorescent dyes such as 2-aminobenzamide (2-AB) and 2-aminobenzoic acid (2-AA). The labeled glycans are then separated using high-performance liquid chromatography (HPLC) or capillary electrophoresis (CE), detected with a fluorescence detector, and identified and quantified by comparing with standards. Alternatively, MS-based analysis of permethylated glycans is also often performed to directly obtain structural information. With instrumental advances, MS-based approaches have become the gold standard of glycan-analysis methods, especially in the field of glycoproteomics [13]. Nuclear-magnetic-resonance (NMR) spectroscopy is also often used to understand the fine details of the glycan structure [14].

For all these glycan-analysis methodologies it is necessary to select appropriate methods for pretreatment and analysis, which depend on the type of glycans being examined. In addition, to understand the functions of the glycans attached to glycoproteins, it is necessary to know which glycan is attached to which glycosylation site on which protein. MS-based approaches allow this site-specific glycoform analysis, which has been particularly successful for the analysis of *N*-glycoproteins [13]. During this analysis, glycans are analyzed without being released from the glycoproteins. This poses a great challenge for this type of analysis due to glycan heterogeneities at the macro level (i.e., the presence or absence of glycosylation at potential glycosylation sites), at the micro level (i.e., the variation of glycoforms for a single glycosylation site), and at the meta level (i.e., the variation of glycoforms for a whole protein) [15]. Due to glycan heterogeneity, the sensitivity of the analysis is crucial for a comprehensive understanding of the glycosylation state of proteins. Usually, such detailed structural analyses require a quantity of analyte on the order of a microgram or more, which may be a critical issue, especially for endogenous biological samples due to limited availability.

## 2 Principle and application of lectin microarrays

**Fig. 2:**
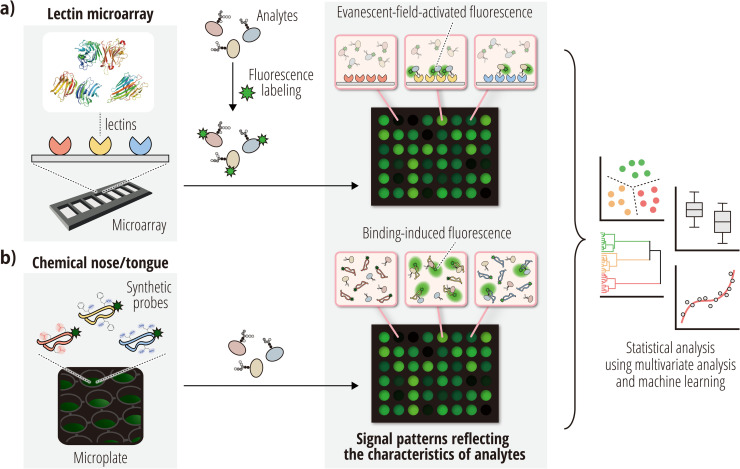
Principles of the lectin microarray and chemical nose/tongue. (a) Schematic diagram of a typical evanescent-field-based LMA. A series of lectins (i.e., sugar-binding proteins) are immobilized on the glass substrate of the microarray. After analytes pre-labeled with a fluorescent marker are applied to the microarray, illumination at a specific angle generates an evanescent field, which selectively excites fluorophores on the labeled analytes bound to the lectins. This allows for the selective detection of fluorescence only from bound analytes. (b) Schematic diagram of a typical fluorescence-based chemical nose/tongue. When analytes are added to a microplate containing aqueous solutions of synthetic probes with different chemical structures and incorporated fluorophores, each probe interacts cross-reactively and differently with the analytes. The strength of these interactions is output as changes in fluorescence. In both approaches, the combination of detected fluorescent responses forms a unique ‘fluorescence pattern’ for each analyte. Finally, the generated patterns are analyzed and compared using multivariate analysis or machine learning.

To overcome the aforementioned issues in the structural analysis of glycans, especially the challenges related to diverse and complex glycan structures, ‘glycan profiling’ approaches have been developed based on interactions between glycans and glycan-binding molecules. So far, the lectin microarray (LMA) is the most successful glycan profiling method, which employs a variety of well-characterized lectins with different carbohydrate specificities as glycan-binding proteins [[16], [17], [18]]. The resulting glycomic profile is based on the patterns of lectin–carbohydrate interactions. Since the first publication by several research groups in 2005 [[19], [20], [21], [22]], the LMA has been used in many studies and is described in over 300 literature reports, and it is now accepted as one of the gold standards of glycan analysis methods. For example, the utility of the LMA for establishing compliance with recombinant therapeutic proteins has been approved by the U.S. Food and Drug Administration (FDA) [23]. To date, LMA platforms have been published by multiple research groups and some of these are now commercially available. Most LMA platforms are slide-format arrays (Fig. 2a), which are typically operated using the following four steps: (i) fabrication of lectin array chips using multiple lectins with different carbohydrate specificities; (ii) reaction of the immobilized lectins with the carbohydrates in the analytes; (iii) data acquisition by measuring the signal intensities corresponding to lectin–carbohydrate interactions; and (iv) data processing to obtain glycomic profiles, which are subsequently used for further data analysis [17].

LMA technology has several advantages compared to other glycan-analysis methods. One notable advantage is that it allows easy comparison of the glycomic profiles obtained from multiple samples of tiny amounts of glycoconjugate samples via simple procedures of sample preparation and analysis. Another advantage is the versatility of the analytes that can be examined using LMA; this differential glycomic-profiling procedure is applicable to various biological sources including crude and purified glycoproteins (endogenous or recombinant), intact cells, microorganisms, extracellular vesicles, body fluids, and tissues [17]. In addition, since lectins consist of a diverse group of glycan-binding proteins, LMA allows the analysis of different glycan categories including *N*-glycans, *O*-glycans, glycolipids, and sulfated GAGs, by employing lectins with affinities to such glycans in the array panel [24]. This technology facilitates a sensitive analysis for glycoproteins on the order of nanograms, which is superior to the other glycan analytical methods introduced above. For example, cell lysates prepared from three cultured cells were sufficient for differential analysis using an ultrahigh-sensitive evanescent-field fluorescence-assisted scanner [25]. Notably, since lectins themselves are functional molecules *in vivo*, LMA can provide information on glycan epitopes such as sialylation, core-fucosylation, and bisecting N-acetylglucosamine (GlcNAc), which are highly relevant in the context of various biological activities and diseases.

Taking advantage of the above features, the LMA is usually used prior to a detailed glycan-structure analysis based on MS to give an overview of the characteristics of the glycans attached to the glycoconjugates, especially glycoproteins. One main objective of LMA analysis is to select suitable lectins for the recognition of the target glycan structures. The selected lectins can then be used for subsequent lectin-based detection and evaluation, such as lectin blotting and lectin histochemistry. The selected lectins can also be used to collect glycoconjugates with the target glycan structures by enrichment from the crude sample, facilitating the subsequent MS-based detailed structural analysis that requires high sensitivity. In addition, the LMA is also used for the verification step, after MS-based carrier-protein identification, by using an antibody of the target glycoprotein (i.e., antibody-overlay LMA). This multimodal glycan analysis strategy that combines lectin-based and MS-based approaches is particularly useful for the discovery of disease-related glycans from clinical specimens that usually require the handling of minute amounts of sample [5,26].

Another notable application of LMAs is ‘tissue glycome mapping.’ This is a spatial glycomics technique for gaining an overview of the quantitative and qualitative variations of glycan structures expressed in each minute area (containing ∼60 cells) of formalin-fixed paraffin-embedded (FFPE) tissue sections [[25], [27]]. Similar to a spatial proteomics technique called deep visual proteomics, laser microdissection is employed for tissue sample collection under microscopic observation, and the resulting tissue fragments are subjected to protein extraction for subsequent LMA analysis [28]. Public tissue glycome mapping data obtained using this method are available for over 500 glycomic profiles and relevant histochemical images for 14 tissues of normal and diseased mice accessible on a database called LM-GlycomeAtlas [29,30].

Although LMAs have distinct advantages as described above and have become one of the most commonly used glycan-analysis methods, there are several limitations pertaining to current LMA technology. Firstly, the repertoire of lectins that can be obtained from natural resources is limited and hence there are limitations on the glycan structures that can be analyzed. It is known that glycans are possibly modified with acetyl, methyl, phosphate, sulfate, and other groups by specific transferases. Such modifications are often not a focus of LMA analysis due to the lack of specific binding probes. This limitation can be partly solved by using other glycan-binding molecules, such as glycan-specific imprinted polymers [31]. Secondly, the LMA analytical procedure consists of multiple steps that must be performed manually, and thus some training is needed to obtain reproducible results. Finally, the results obtained from LMA analysis are also affected by the lot-to-lot variations of the microarray chips caused by the lectins and the fabrication processes, which leads to a limitation of the number of samples that can be simultaneously analyzed. Although still in its infancy and not yet proven to have the same applicability as LMAs, the following ‘chemical-nose/tongue’ technology may complement and overcome some of the limitations associated with LMA analysis.

## 3 Principle of chemical noses/tongues

The chemical nose/tongue is an analytical technology modeled on the function of human smell and taste. This technology evaluates samples by recognizing ‘optical patterns’ generated through cross-reactive interactions with synthetic probes [[32], [33], [34], [35]]. A typical chemical-nose/tongue system combines a set of chromogenic or fluorogenic synthetic probes to obtain an optical output that is then examined using multivariate analysis or machine-learning algorithms. The analytical process begins with the preparation of an array of solutions, each containing various synthetic probes with different chemical structures (Fig. 2b). Upon adding the sample, each probe interacts differently with its components, outputting these interactions as changes in the absorbance or fluorescence spectra. The collective responses form an optical pattern unique to the sample. The resulting optical patterns are then analyzed and compared using multivariate analysis or machine-learning techniques.

The chemical-nose/tongue strategy offers several distinct advantages. As the probes are synthetically produced, their target-recognition sites and signal-output mechanisms can be designed arbitrarily. This flexibility facilitates easy expansion and improvement of the synthetic probe repertoire, allowing the detection format and procedure to be tailored to specific applications. In addition, the synthetic probes do not need to be immobilized, and the system can be constructed in a simple ‘mix-and-read’ format, enhancing ease of use and adaptability. Similar to LMAs, chemical-nose/tongue systems collect multidimensional information from the sample through cross-reactive responses. However, there is a crucial difference in approach. Particularly when applied to complex biological samples, chemical-nose/tongue systems are often referred to as ‘hypothesis-free sensor arrays’ [36,37]. This is because they do not require prior knowledge of how the probes interact with the specific sample components to construct the system. In such cases, the primary goal is often to recognize differences, rather than to pinpoint specific marker molecules. This hypothesis-free approach marks a significant departure from LMA analysis, which typically relies on well-characterized binding pairs.

While LMA and chemical-nose/tongue systems differ in their design flexibility and applications, the foundational concepts share remarkable similarities. Nevertheless, these technologies have historically evolved along different developmental trajectories. The concept of the chemical nose/tongue can be traced back to the 1980s, originating from the pioneering work of Persaud and Dodd [38]. Their research laid the groundwork for what would eventually become known as the ‘electronic nose/tongue’. The electronic nose/tongue detects electrical patterns generated through the adsorption of sample components onto sensor arrays made of materials like metal-oxide semiconductors [39]. The evolution of this concept took a significant leap forward in the 2000s when Suslick and colleagues extended this concept to chemical sensors [32,40], laying the foundation of the chemical nose/tongue. This development was further advanced in the late 2000s by Rotello and colleagues, who explored the potential of applying chemical-nose/tongue systems to a diverse range of biological samples [33,41,42].

Since then, the versatility of chemical-nose/tongue systems has been demonstrated through their application to a wide spectrum of biological samples, including metabolites [[43], [44], [45], [46]], proteins [[47], [48], [49], [50], [51], [52]], DNA/RNA [[53], [54], [55]], biofluids [[56], [57], [58], [59], [60], [61]], cells [[62], [63], [64], [65], [66], [67], [68], [69], [70]], microorganisms [[71], [72], [73], [74], [75], [76], [77], [78]], and beverages [36,[79], [80], [81], [82], [83]]. Notably, these applications also extend to the analysis of monosaccharides and glycans. In the following sections, we will present the specific applications of chemical-nose/tongue systems that target monosaccharides and glycans. By examining the examples reported to date, we aim to illustrate the unique capabilities of this technology in glycan analysis and its potential to advance our understanding of glycobiology and related fields.

## 4 Chemical noses/tongues targeting saccharides

### 4.1 Utilization of boronic acids

**Fig. 3:**
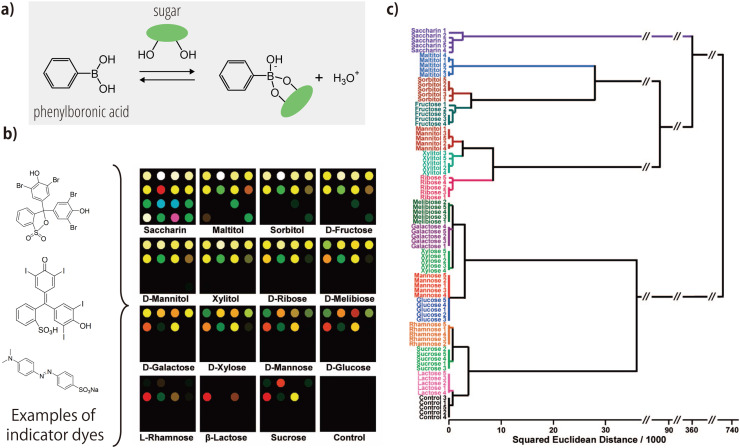
Saccharide recognition using color changes of indicator dyes mediated by the reactions between boronic acid and saccharides. (a) Reaction scheme showing the formation of a cyclic boronate ester from phenylboronic acid and a saccharide. (b) Color-difference maps illustrating the changes in the colors of the indicator dyes after saccharide addition, resulting in unique patterns for each saccharide and sweetener. (c) The resulting dendrogram obtained by hierarchical clustering analysis of the 15 saccharides and sweeteners (25 mM), except for sucrose (150 mM). Adapted with permission from Ref. [88]. Copyright 2008 American Chemical Society.

Chemical-nose/tongue systems for the analysis of saccharides have predominantly relied on boronic-acid chemistry, often referred to as ‘boronlectins' [[84], [85], [86]]. The foundation of this approach lies in the unique ability of boronic acids to form cyclic boronic esters by forming dynamic covalent B-O bonds with *cis*-1,2-diols or 1,3-diols, structural features that are prevalent in saccharides (Fig. 3a). Early saccharide-recognition studies using chemical-nose/tongue strategies leveraged the color changes of indicator dyes, caused by the formation of cyclic boronic esters, as pattern information for saccharide differentiation [87,88]. The mechanism behind these color changes relies on two factors: (i) the color of the indicator dyes is influenced by decreases in pH value, due to the hydronium ions generated during the reaction between the arylboronic acids and saccharides; (ii) various cross-reactive factors, including Lewis acid/base interactions and hydrogen bonding between the saccharides (or their adducts) and the dyes, contribute to the unique color patterns observed.

A notable example of this approach is the work of Lim et al. [88], who employed a combination of 3-nitrophenylboronic acid and 16 different indicator dyes to generate unique color patterns for monosaccharides, disaccharides, sugar alcohols, and artificial sugars present at concentrations on the order of tens of mM (Fig. 3b). To analyze the resulting patterns, the colors were converted into RGB values and processed using hierarchical cluster analysis, a representative multivariate-analysis technique (Fig. 3c). The resulting dendrogram indicated that all 15 saccharides and sweeteners could be accurately distinguished.

**Fig. 4:**
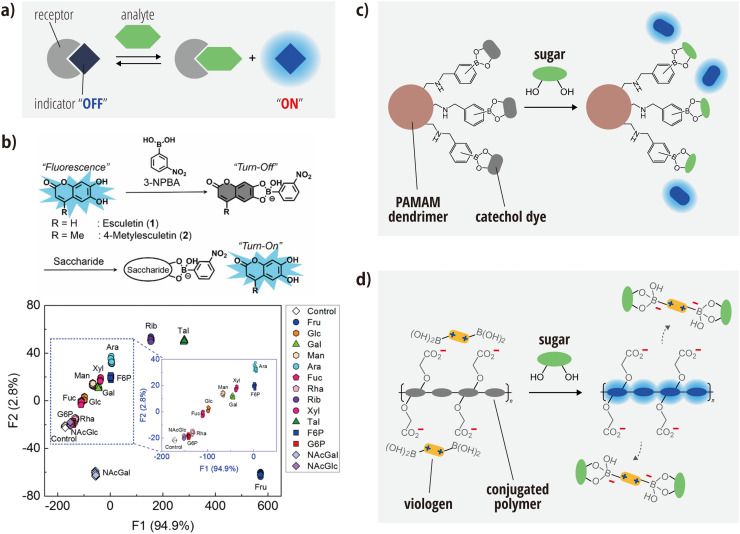
Saccharide recognition based on indicator-displacement assays and boronic-acid chemistry. (a) Conceptual illustration of the indicator-displacement assay. The optical state of the indicator changes when displaced by the analyte. (b) Saccharide recognition using catechol adducts. The 3-nitrophenylboronic-acid (3-NPBA) adduct of a catechol fluorophore produces a fluorescent response upon displacement by a saccharide (top). Plot of linear-discriminant scores obtained from the analysis of fluorescence patterns of saccharides at a concentration of 6 mM (bottom). Adapted with permission from Ref. [91]. Copyright 2019 American Chemical Society. Schemes showing indicator-displacement assays for saccharide recognition utilizing (c) dendrimers bearing phenylboronic acid with attached catechol fluorophores and (d) anionic conjugated polymers quenched via the formation of a complex with cationic benzyl viologens.

The indicator-displacement-assay format has emerged as a widely adopted strategy for the construction of chemical-nose/tongue systems for saccharide recognition. Its popularity stems predominantly from its ability to offer a flexible design that generates both optical responses and diverse recognition capabilities (Fig. 4a). In a typical indicator displacement assay, the indicator dye is reversibly bound to the receptor either covalently or non-covalently. Upon the introduction of a sample, the dye is displaced, producing an detectable optical signal (e.g., absorbance or fluorescence) [89].

The use of a covalent indicator displacement assay using conjugates of catechol dyes and arylboronic acids is representative of the approaches used in this field [[90], [91], [92], [93], [94]]. For example, Sasaki et al. have constructed a system in which 3-nitrophenylboronic acid binds to a fluorescent coumarin derivative with a diol group (Fig. 4b, upper panel) [91]. In the bound state, the fluorescence of the coumarin derivative is quenched via a photoinduced electron transfer (PeT) mechanism. The addition of a saccharide displaces the coumarin derivative, restoring fluorescence. The ingenuity of this work lies in the utilization of the pH-dependent differences in the cross-reactivity between the two coumarin derivatives. This allowed the researchers to achieve highly accurate discrimination of 14 saccharides at a concentration of 6 mM. The discrimination was based on the absorbance spectra patterns measured under different pH conditions. The lower panel of Fig. 4b shows the discrimination scores obtained through linear-discriminant analysis. The non-overlapping clusters for each saccharide suggest statistically significant differences among the patterns. Indeed, 100 % discrimination accuracy was afforded through cross-validation tests. This system was also able to both quantify mixtures of three saccharides using machine learning and monitor saccharide consumption by human iPS cells in culture media. Another similar covalent chemical-nose/tongue system involves fluorescent catechol dyes attached to poly(amidoamine) (PAMAM) dendrimers with terminal phenylboronic-acid groups (Fig. 4c) [95].

Noncovalent-indicator-displacement assays have also been used to construct chemical-nose/tongue systems for saccharide recognition. These assays often use complexes formed through electrostatic interactions between cationic molecules functionalized with phenylboronic acid (e.g., viologens possessing multiple pyridyl groups) and anionic fluorescent molecules [[96], [97], [98], [99]]. The standard mechanism for these assays is as follows: the anionic fluorescent molecule is quenched when bound to the boronic-acid-appended viologens. When a saccharide reacts with the boronic acid to form a complex, it becomes negatively charged due to a decrease in pKa. Consequently, the formed complex dissociates, leading to the restoration of fluorescence. One exemplary approach employs a conjugated polymer with carboxyl groups (Fig. 4d) [99]. A more recent noncovalent approach uses luminescent lanthanide metallogels quenched by the loading of 3-nitrophenylboronic acids [100].

More direct strategies that do not rely on indicator displacement have also been developed. One example is a chemical nose/tongue using bispyridinium functionalized with phenylboronic acid and fluorescent pyrene [101]. The operating principle of this system is based on saccharide-induced aggregation. When the phenylboronic acid binds to the saccharides, the pyrene moieties aggregate, causing excimer emission at different wavelengths. In this study, the use of two bispyridinium derivatives enables the discrimination of six different saccharides at a low concentration (100 μM).

### 4.2 Other examples

**Fig. 5:**
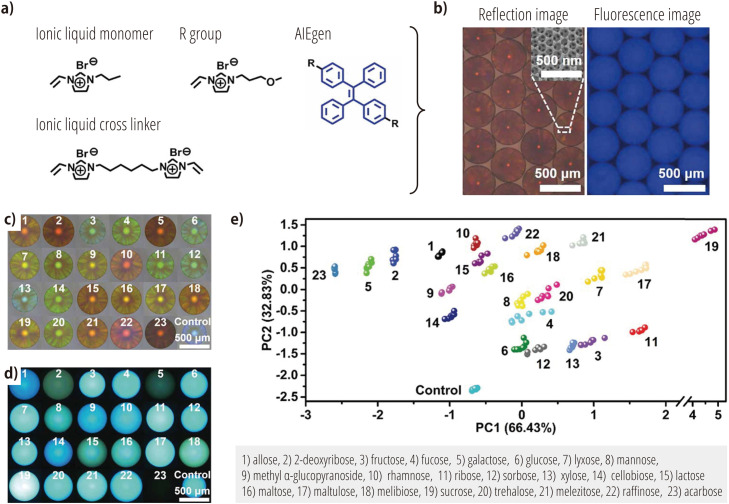
Saccharide recognition using poly(ionic liquid) spheres. (a) Chemical structures of the ionic-liquid monomer, crosslinker, and aggregation-induced-emission (AIE) luminogen used as raw materials for sphere synthesis. (b) Reflection and fluorescence images of the poly(ionic liquid) spheres doped with the AIE fluorophores. Inset: scanning-electron-microscopy images. (c) Reflection images and (d) fluorescence images of the spheres after exposure to buffered aqueous solutions (pH = 7.0) of 23 different sugars at 100 mM. (e) Plot of the principal-component scores obtained from the analysis of the photonic and fluorescence properties of the spheres in response to various saccharides. Reproduced from Ref. [102] with permission from the Royal Society of Chemistry.

While boronic-acid chemistry has dominated the field of saccharide recognition in chemical-nose/tongue systems, approaches that utilize alternative mechanisms have also been developed. For example, Zhang et al. have developed a system composed of poly(ionic liquid) spheres doped with fluorophores that exhibit aggregation-induced-emission (AIE) properties [102]. The fabrication of these photonic spheres involves a three-step process: (i) monodisperse silica nanoparticles are self-assembled to form photonic spheres with an ordered lattice structure. (ii) Ionic liquid monomers, cross-linkers, and AIE fluorophores (Fig. 5a) are then allowed to penetrate the interstitial spaces of the photonic spheres where they are polymerized. (iii) The silica template is removed using hydrofluoric acid. The resulting inverse opal-like photonic spheres possess interconnected pore structures (Fig. 5b, inset) and exhibit both photonic properties, arising from the periodic porous structure, and fluorescence, originating from the doped AIE fluorophores (Fig. 5b). This dual-mode optical response forms the basis of the system. Upon exposure to saccharides, the spheres experience multiple noncovalent interactions, including hydrogen bonding and hydrophobic interactions. These interactions lead to changes in the refractive index due to expansion or contraction of the photonic spheres (Fig. 5c) and changes in fluorescence intensity of the AIE fluorophores due to variations in the local environmental (Fig. 5d). Using this dual-response mechanism, it was possible to differentiate 23 different saccharides at a concentration of 100 mM (Fig. 5e), with a 100 % discrimination accuracy through cross-validation tests, which highlights the powerful performance of this method.

Recent developments have further expanded the range of available methods. One approach employs the glucose oxidase-like catalytic activity of gold nanoparticles [103]. This method relies on the detection signals based on colorimetric changes associated with the reduction of various dyes coupled with saccharide oxidation by gold nanoparticles.

By using different types of reactions, such as specific covalent bonding with boronic acids or less specific noncovalent interactions, it is possible to construct chemical-nose/tongue systems for saccharide recognition. The choice of detection mechanism significantly influences the applicability and performance of the systems. Notably, boronic-acid-based approaches, due to their specificity, enable saccharide detection in complex matrices, including cell culture media [91], urine [92], and fruit beverages [93]. Thus, these strategies hold promise for analyzing real samples containing free saccharides, whether produced enzymatically or non-enzymatically.

## 5 Chemical noses/tongues targeting glycans

### 5.1 Glycoaminoglycans

Chemical noses/tongues now have the capability to detect polysaccharides, especially GAGs [104]. GAGs are a family of linear, highly negatively charged polysaccharides essential for numerous biological processes, including the control of cell properties, tissue development, homeostasis, and disease progression [105]. Due to their significant biological importance, GAGs have garnered significant interest in the context of biomedical applications. Examples include heparin (used as an anticoagulant in surgeries and hemodialysis) [106], chondroitin sulfate (CS; used for alleviating inflammation and pain in osteoarthritis) [107], and hyaluronic acid (HA; used in ophthalmic and orthopedic surgeries, as well as for skincare) [108]. However, the chemical structure of GAGs is complex and highly heterogeneous due to the biosynthetic process by which they are produced, thus raising safety concerns for their clinical use. The critical importance ensuring the purity of GAGs was starkly highlighted during a 2007–2008 incident, where the contamination of the heparin supply chain with over-sulfated CS (OSCS) resulted in multiple fatalities [109,110]. Furthermore, the analysis of urinary GAG profiles is crucial for diagnosing mucopolysaccharidoses, a group of disorders that cause progressive tissue damage across multiple organs [111]. Thus, detecting and identifying GAGs are essential for clinical applications.

While advanced analytical techniques, such as those based on NMR, HPLC, and MS are currently employed for GAG analysis [109], these methods are often costly and require complex procedures. Consequently, there is a pressing demand for the development of simpler and more reliable methods for GAG analysis. Chemical-nose/tongue systems potentially offer a solution to this challenge. So far, these systems are designed primarily to recognize the physicochemical properties of glycans, leveraging their hypothesis-free nature that allows for comparative analysis based solely on response patterns.

**Fig. 6:**
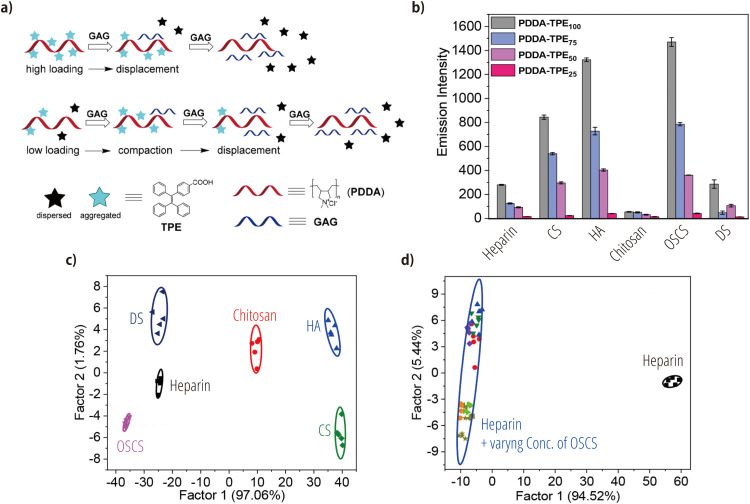
GAG recognition using AIE nano-assemblies. (a) Schematic illustration of the recognition mechanism based on indicator-displacement assays. Top: at high loading, PDDA bearing TPE derivatives releases quenched TPE derivatives upon displacement through interaction with GAGs. Bottom: at low loading, GAG binding induces further quenching of the TPE derivatives prior to their release. (b) Fluorescence patterns observed when GAGs (51.2 μg/mL) are mixed with PDDA/TPE nano-assemblies consisting of various loading ratios. (c) Plots of linear discriminant scores obtained from the analysis of (c) fluorescence patterns of GAGs at 51.2 μg/mL and (d) fluorescence patterns of heparin (Hep) contaminated with varying concentrations of OSCS, maintaining a total concentration of 51.2 μg/mL. Adapted with permission from Ref. [112]. Copyright 2019 American Chemical Society.

Most reported examples of chemical noses/tongues for GAG analysis are based on indicator-displacement assays. Yang et al. have used supramolecular complexes formed between cationic poly(diallyldimethylammonium chloride) (PDDA) and an anionic carboxyl-modified AIE luminogen, specifically a tetraphenylethene (TPE) derivative [112]. When these molecules are mixed, multiple TPE derivatives are carried by the PDDA, enhancing fluorescence due to the restricted rotational motion of the TPE derivatives (Fig. 6a). Upon subsequent addition of negatively charged GAGs (heparin, CS, HA, OSCS, or dextran sulfate (DS)), the GAGs displace the TPE derivatives, which leads to fluorescence quenching (Fig. 6a, upper panel). If the loading of the TPE derivatives on the PDDA is low, fluorescence enhancement occurs prior to displacement. This is due to compression caused by the formation of a ternary complex (Fig. 6a, middle panel). Using these phenomena, a chemical-nose/tongue system with supramolecular complexes of varying TPE loadings was constructed. This system generated characteristic fluorescence patterns for six different GAGs, including chitosan (51.2 μg/mL) (Fig. 6b). Linear-discriminant analysis demonstrated the ability to cluster all the GAGs (Fig. 6c), achieving 100 % accuracy in cross-validation tests. Furthermore, the system successfully distinguished heparin samples contaminated with 1–50 w/w% OCSC from pure heparin (Fig. 6d), highlighting its potential for quality control in heparin products.

Various other fluorescent chemical-nose/tongue systems based on indicator-displacement assays for GAG recognition have also been reported (Table 1). However, not all effective systems rely on the displacement phenomenon. One example that deviates from the displacement-based approach involves the use of nanosized aggregates of structurally different cationic conjugated polymers [113]. In this system, the addition of GAGs induces changes in the aggregation state of the polymers, resulting in alterations of the fluorescence signals.

**Table 1:** Chemical-nose/tongue approaches for GAG recognition via indicator-displacement assays.

Probe complexes	Conditions for GAG identification		Other analyses	Refs.
	Selected GAGs	Conc.		
Cationic cyclodextrin derivatives and fluorescent reporters	UFH, LMWH, HS, DeS, CS, OSCS	6 μg/mL	2–3 Glycan mixtures	[114]
Fe(II), amine-functionalized dipicolylamin, Evans blue dye	LMWH, UFH, DS, CS, DS, HS	25 μg/mL	• 6 Glycans at 10, 25, 50 μg/mL • 2 Glycan mixtures	[115]
Cationic gold nanoparticles and anionic conjugated polymers	Heparin and its derivatives, chitosan, HA, 2 types of CS, 3 types of DS	N/A	• Sulfonation and acetylation • Differences in size and charge • Epimeric nature	[116]
Cationic dendrigrafts and carboxyfluorescein-modified tri-aspartic acids	Heparin, 2 types of CS, HA, DS	3.5 mg/mL	2 Glycan mixtures	[117]
Cationic cyclic calixarene hosts and eosin Y dye	Heparin, HA, DS, 3 types of CS	0.8 μM		[118]
Cationic porphyrin and anionic graphene oxide	Heparin, CS, HA, DS	50 μg/mL	• 100 μg/mL Glycan in 10 % serum • 2 Glycan mixtures	[119]
Cationic porphyrin and carboxyl-modified pyrene	Heparin, CS, HA, DS	10 μg/mL	5 μg/mL glycan in 5 % serum	[120]

Abbreviations: unfractionated heparin (UFH); low-molecular-weight heparin (LMWH); heparan sulfate (HS); dermatan sulfate (DeS).

A key feature common to all the above examples is that the responses for differentiating GAGs are primarily generated through nonspecific interactions between the GAGs and the probes. This characteristic underscores one of the fundamental strengths of the chemical-nose/tongue strategy: its hypothesis-free nature in sensing-system construction. This recognition mechanism provides a flexible platform that can be adapted to detect a wide range of GAGs without the need for developing specific probes for each target. Moreover, it can potentially capture subtle differences between GAGs that might be overlooked. Such strategies, distinct from conventional methods, may open up new possibilities to facilitate the clinical use of GAGs.

### 5.2 Glycoproteins

Protein glycosylation is the most abundant post-translational modification in eukaryotic cells and plays a crucial role in cell functions and disease processes. The enzymatic glycosylation of proteins involves complex metabolic networks and various glycosylation pathways, resulting in immense structural diversity. This diversity poses analytical challenges that hinder the exploration of the specific functions of glycoproteins [121]. Glycoproteins have also become key targets for chemical-nose/tongue systems.

In the development of chemical-nose/tongue systems for glycoprotein analysis, a key challenge is to prioritize glycan-related information while minimizing the influence of the protein scaffold. To address this, boronic-acid chemistry has been chosen. For example, fluorescent quantum dots functionalized with phenylboronic acid can bind to glycoproteins via reactions with *cis*-diols, thereby generating distinctive optical-signal patterns characteristic of glycoproteins [122]. Another recently published system employs complexes of phenylboronic-acid-decorated carbon dots and arginine-modified titanium carbides [123]. This approach has shown remarkable sensitivity for the detection of glycoprotein messenger cytokines, such as interleukin 6 (IL-6), IL-8, and tumor necrosis factor-α (TNF-α), in saliva at 20 ng/mL. Notably, removing the phenylboronic-acid moieties significantly decreased the accuracy, suggesting that recognition of the glycan moieties contributes to this high performance.

**Fig. 7:**
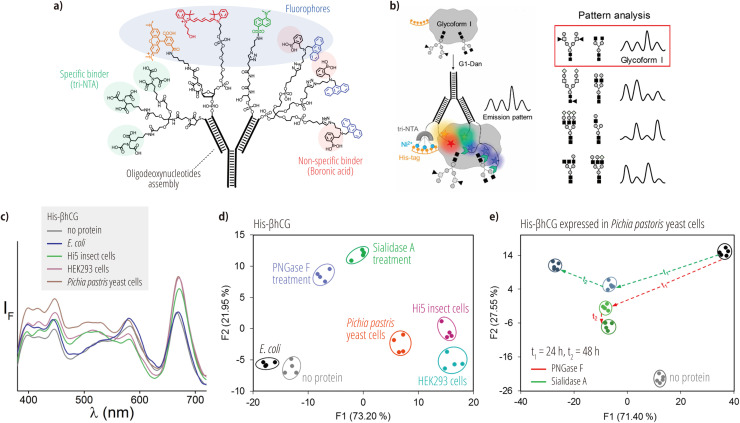
Protein-glycoform recognition using self-assembled sensors. (a) Schematic illustration of the self-assembled sensor. The sensor consists of four oligodeoxynucleotides modified with His-tag-specific binders (tri-NTA units), non-specific glycan binders (anthracene/boronic-acid units), and various fluorescent reporters. (b) Schematic illustration of the glycoform recognition principle used in the self-assembled sensor. (c) Emission spectra of the self-assembled sensor in the presence of various His-βhCGs (900 nM). Plots of linear-discriminant scores obtained from the analysis of (d) various His-βhCGs and (e) His-βhCG expressed in yeast cells incubated with two different glycan-cleaving enzymes for various periods of time. Adapted with permission from Ref. [124]. Copyright 2020 American Chemical Society.

Peri-Naor et al. have developed an innovative approach for differentiating between distinct glycoforms on specific protein surfaces using supramolecular complexes formed via DNA hybridization [124]. This method employs four functionalized oligodeoxynucleotides, each functionalized with three key components: A His-tag-specific binder (trinitrilotriacetic acid (tri-NTA)), a nonspecific glycan binder (fluorogenic anthracene-boronic acid), and various fluorescent reporters (Fig. 7a). Binding between the supramolecular complexes and glycoproteins via the tri-NTA and glycan binders alters the local environment of the fluorescent reporters. This leads to complex emission patterns due to a combination of various photophysical and photochemical processes (Fig. 7b). Crucially, differences in the glycoforms lead to varying affinities and local-environmental changes in the supramolecular complex, producing unique emission patterns specific to each glycoform (Fig. 7b).

The glycoforms of His-tagged human chorionic gonadotropin β subunits (His-βhCG) vary depending on the host used for expression (e.g., insect, human, or yeast cells), while *E. coli* produces nonglycosylated His-βhCG. The supramolecular complex displays complex emission patterns that effectively captured these differences (Fig. 7c). Linear-discriminant analysis enables the discrimination of these glycoforms as well as the detection of enzymatic glycan cleavage (Fig. 7d). Furthermore, this system demonstrated the ability to sense the progression of glycosidase-mediated cleavage (Fig. 7e). Glycosylation is a critical attribute of therapeutic proteins, including antibodies, and plays a key role in determining the efficacy, safety, and pharmacokinetic properties of biologics [125]. Therefore, this technology holds potential for use as a complementary method for quality control in biopharmaceutical manufacturing processes.

### 5.3 Glycomic signatures of cell surfaces

Cell-surface glycans play a central role in numerous biological processes, including cell-cell recognition, inflammation, immune surveillance, pathogenicity, and viral recognition. These glycans undergo significant changes depending on the cell state (e.g., cell differentiation, tissue development, and cancer progression) [[126], [127], [128]]. Consequently, glycan profiling is recognized as a powerful means for detecting malignant cells, predicting cell states and behavior, and developing therapeutic agents [129,130]. However, the inherent complexity of glycan structures renders their analysis challenging. Chemical-nose/tongue systems have the potential to address these challenges related to cell-surface glycan recognition.

Rotello and colleagues have made significant contributions to this field. They initially designed a chemical-nose/tongue system using complexes consisting of cationic gold nanoparticles and three differently colored anionic fluorescent proteins [131]. This system successfully distinguished between a set of GAG-mutated Chinese hamster ovary (CHO) cell types and glycosidic enzyme-treated wild-type CHO cells.

**Fig. 8:**
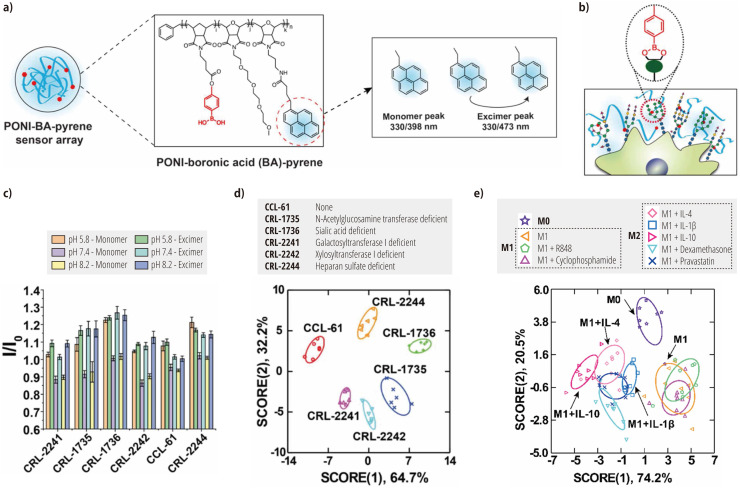
Recognition of glycomic signatures of cell surfaces using fluorescent polymers. (a) Chemical structures of PONI random copolymers functionalized with phenylboronic acid, tetra(ethylene glycol), and pyrene. Boronic acid serves as the recognition element, while pyrene serves as the transducer, exhibiting a monomer emission of 398 nm and an excimer emission of 473 nm when excited at 330 nm. (b) Schematic illustration of the interactions between the sensor elements and cell-surface glycans. (c) Fluorescence patterns observed when CHO glycosylation mutants were mixed with the PONI-based polymer. Plots of linear-discriminant scores obtained from the analysis of (d) the fluorescence patterns of CHO glycosylation mutants and (e) M1 macrophages after treatment with different drugs. Panels a–d: Reproduced from Ref. [132] with permission from the Royal Society of Chemistry. Panel e: Adapted with permission from Ref. [133] Copyright 2023 American Chemical Society.

Building on this work and aiming for more direct glycan detection, the Rotello group subsequently developed a polymer-based system incorporating boronic acid [132]. This system utilizes a poly (oxanorborneneimide) (PONI) random copolymer. The polymer is functionalized with three components: phenylboronic acid for glycan recognition, tetra(ethylene glycol) to improve the polymer solubility, and pyrene for transducing interactions into fluorescent signals (Fig. 8a). Pyrene units form excimers when in close proximity to one another, which emit longer-wavelength fluorescence, thus contributing to the generation of diverse responses. The PONI-based polymers bind mainly to diols on the cell surface, and this interaction is converted into pyrene fluorescence emission (Fig. 8b).

To demonstrate the capability of the system, the researchers adopted an approach of mixing aqueous solutions of PONI-based polymers with cells under various pH conditions. This approach enabled the generation of fluorescence patterns specific to CHO glycomutants (Fig. 8c). Subsequent analysis of these patterns using linear-discriminant analysis produced distinct, non-overlapping clusters (Fig. 8d) with 100 % discrimination accuracy.

The versatility of the PONI-based approach has been further demonstrated by using it to monitor drug-induced macrophage repolarization [133]. The extension of the technology is particularly significant given that macrophage polarization and function are closely related to changes in cell-surface glycosylation [134]. Macrophages are key players in the innate immune system and are capable of polarizing into multidimensional phenotypes in response to different stimuli. The two major phenotypes of polarized macrophages are the inflammatory M1 and anti-inflammatory M2 states. Controlling macrophage polarization is expected to provide therapeutic strategies for various diseases, including autoimmune and inflammatory conditions [134]. Rotello and colleagues have selected the mouse-derived macrophage cell line RAW 264.7, which is polarized from M0 to M1 using lipopolysaccharides, as a model target for their chemical-nose/tongue system. To explore the ability of the system, the M1-polarized macrophages were exposed for 48 h to a range of substances: (i) two drugs (dexamethasone and pravastatin) and three cytokines (IL-4, IL-1β, and IL-10) known to induce M2 repolarization, and (ii) two drugs (R848 and cyclophosphamide) that promote M1 polarization. The fluorescence patterns generated by mixing these macrophages with PONI-based polymer solutions differentiated the expected effects of the drugs, thereby distinguishing the M0, M1, and M2 states (Fig. 8e).

These findings highlight the power of chemical noses/tongues for targeting cell-surface glycans. Such approaches cannot only detect intrinsic differences in cell states but also extrinsic differences resulting from external stimuli, such as drug exposure. In particular, the ability to rapidly identify macrophage repolarization in response to drug treatment offers significant advantages in pharmaceutical research for enhancing drug-efficacy evaluation and accelerating drug discovery.

## 6 The power and potential of chemical noses/tongues in comparison to LMA

In this review we have focused on chemical-nose/tongue technology and the promise which it holds as a new technology for glycan analysis, discussing its principles and showcasing various applications for monosaccharide and glycan detection. This analytical technology enables efficient profiling of monosaccharides and glycans by generating multivariate data through cross-reactive synthetic-probe sets and processing the data using statistical techniques. To assess how this technology complements and potentially stands alongside LMAs, one of the current gold standards in glycan analytical methods, we will now outline the similarities and differences between the two technologies and comprehensively discuss the current position and future potential of chemical-noses/tongues in the field of glycan analysis.

### 6.1 Similarities

(i) **Shared foundational concept:** Both chemical noses/tongues and LMAs are based on a similar core idea, i.e., comprehensively profiling interaction patterns between multiple recognition probes and the target samples. By focusing on the overall interaction profile rather than individual molecular interactions, both technologies enable efficient characterization of complex glycan structures.
(ii) **Simplicity in sample handling:** The recognition probes (synthetic molecules for chemical noses/tongues and lectins for LMAs) can directly interact with accessible sample components, such as those on cell surfaces. This feature often eliminates the need for extensive sample preprocessing, allowing both technologies to be applied directly to a wide range of biological resources, including proteins, intact cells, microorganisms, extracellular vesicles, biofluids, and tissues.
(iii) **Customizability:** Both technologies allow for customization by optimizing the combination of recognition probes from their repertoires based on the specific properties of the targets. This flexibility enables the creation of tailor-made array panels designed to address particular glycan categories or research objectives.

### 6.2 Differences

(i) **Range of detectable structures:** LMAs rely on naturally derived lectins, which inherently limits their repertoire. This restriction can make it challenging to detect certain glycan structures, such as those modified by transferases. In contrast, chemical noses/tongues offer the flexibility of designing synthetic recognition probes, which is a significant advantage. For instance, multifunctional probes can be created that combine the specific detection of saccharide moieties using boronic-acid chemistry with capabilities to interact through electrostatic, hydrophobic, and hydrogen-bonding interactions. This enables the detection of glycan structures and enzymatic modifications that are difficult to distinguish using conventional LMAs.
(ii) **Applications:** LMAs are not limited to mere sample differentiation, but can also be used for specific, targeted analysis. After profiling of the samples, specific lectins responsible for the differences can be selected for further in-depth analysis. This approach not only allows for integration with existing scientific knowledge, but also enables the use of selected lectins for applications, such as the enrichment and identification of glycoconjugates carrying target glycans. In contrast, chemical noses/tongues are often employed in a hypothesis-free manner (or non-targeted analysis). While this pattern-based approach may not be ideal for discovering probes that recognize specific glycan structures, it excels in uncovering unknown glycan profiles and detecting broader differences.
(iii) **Other factors:** Chemical noses/tongues provide flexibility in designing both target-recognition sites and signal-output mechanisms, which is helpful for the construction of simple and convenient measurement systems. Another major advantage is the ease of quality control of the system, as synthetic probes are used. In contrast, LMAs rely on biologically derived lectins, which can pose challenges in terms of quality control and structural stability.

### 6.3 Perspectives

Building on the similarities and differences between chemical noses/tongues and LMAs, we discuss here the complementary role of chemical noses/tongues, as well as and the improvements and investigations required for their further development.

(i) **Applications:** While a chemical nose/tongue is not ideal for the precise identification of the types of saccharide units or their sequences, it shows great potential in applications focused on comparing overall glycan structures and composition. For instance, as demonstrated by the recognition of GAGs [112] and protein glycoforms [124] (Fig. 6, Fig. 7), a chemical nose/tongue could be used for advanced quality control in medical applications, such as determining whether a product meets quality standards (e.g., contamination checks or ensuring proper glycosylation). Moreover, as seen in the case of cell-surface glycan recognition [132,133] (Fig. 8), the ability to differentiate cell-surface glycans altered by endogenous or exogenous factors could prove valuable for accelerating biological research and drug discovery. However, whether chemical noses/tongues truly have the potential to capture more detailed information, such as specific glycan epitopes, remains to be explored and will require further investigation with carefully designed sample settings.
(ii) **Formats:** As shown by the example of saccharide recognition using indicator-dye sets [88] (Fig. 3), chemical noses/tongues face fewer constraints in system construction compared to LMAs. This flexibility allows for simplified formats, such as test strips, which could dramatically improve cost-effectiveness and throughput without the need for specialized equipment. However, ensuring that such simplified formats maintain the expected sensitivity and accuracy is a critical area for future evaluation as chemical-nose/tongue technology moves toward practical applications.
(iii) **Sensitivity:** Currently, LMAs have a sensitivity advantage over chemical-nose/tongue systems. LMAs using an ultrahighly sensitive evanescent-field fluorescence-assisted scanner have achieved differential analyses with lysates prepared from as few as three cultured cells [25]. While systems using electrochemical signals have been reported to differentiate samples from fewer than 100 cells [65], typical optical-signal-based chemical-nose/tongue systems require around 1000 cells for analysis [63]. Nevertheless, there remains significant potential for improving the sensitivity of chemical-nose/tongue systems. Exploring new approaches, such as combining it with advanced cellular analysis platforms [72], may enable the achievement of sensitivity comparable to LMA.
(iv) **Further development of both technologies:** The use of synthetic approaches based on molecularly imprinted polymers (MIPs) holds promise. In MIPs, the target molecule (template molecule) is added during polymer synthesis, and after polymerization, the template is removed, leaving binding sites that are specific to the target molecule. MIP technology could help address the limitation of the LMA repertoire and enhance the specificity of chemical noses/tongues. Indeed, MIPs have been used as an approach to efficiently construct chemical-nose/tongue systems tailored to specific molecular categories [135,136], and recent studies have shown that MIPs can specifically recognize subtle differences in glycan structures that lectins cannot detect [31]. MIPs, which represent a conceptually intermediate approach between LMAs and chemical noses/tongues, may expand the capabilities of both technologies for glycan analysis. Furthermore, LMAs could broaden their glycan-detection capabilities by expanding the lectin repertoire using random mutations [137], taking inspiration from the hypothesis-free approach of chemical noses/tongues.

## 7 Conclusion

In summary, chemical noses/tongues hold the potential to address many unresolved niche areas in glycan analysis. By overcoming the current challenges and achieving the necessary performance, this technology could establish itself in the near future as a powerful tool that complements LMAs. The development of chemical noses/tongues would enable more comprehensive and specific glycan profiling, leading to significant breakthroughs in basic biology, medical diagnostics, and drug discovery. Collaboration with glycan-analysis experts will accelerate innovation in chemical noses/tongues, paving the way for its broader applications across various fields.

## CRediT authorship contribution statement

**Shunsuke Tomita:** Conceptualization, Funding acquisition, Writing – original draft, Writing – review & editing. **Chiaki Nagai-Okatani:** Conceptualization, Funding acquisition, Writing – original draft, Writing – review & editing.

## Declaration of competing interest

The authors declare that they have no known competing financial interests or personal relationships that could have appeared to influence the work reported in this paper.

## Data Availability

No data was used for the research described in the article.
